# An appraisal of the neglected tropical diseases control program in Cameroon: the case of the national program against onchocerciasis

**DOI:** 10.1186/s12889-017-4037-x

**Published:** 2017-01-21

**Authors:** Tsi Njim, Leopold Ndemnge Aminde

**Affiliations:** 1 Centre for Global Health and Tropical Medicine, Nuffield Department of Medicine, University of Oxford, Oxfordshire, UK; 2 Health and Human development (2HD) Research Group, Douala, Littoral Cameroon; 3 Clinical Research Education, Networking and Consultancy (CRENC), Douala, Littoral Cameroon; 4 School of Public Health, Faculty of Medicine & Biomedical Sciences, University of Queensland, Brisbane, QLD Australia

**Keywords:** Onchocerciasis, Neglected tropical diseases, Ivermectin, Sustainable development goals, Cameroon

## Abstract

**Background:**

Onchocerciasis is a severe parasitic infestation which causes disabling skin and subcutaneous tissue changes. Current global estimates suggest that it accounts for 1135.7 disability adjusted life years (DALYs) per 100,000 population. The disease is endemic in many African countries including Cameroon, probably suggesting that the current health policies are inadequate to achieve eradication of the disease. We aimed to appraise the current Onchocerciasis control program in Cameroon in the context of existing literature.

**Methods:**

We carried out a MEDLINE search via PubMed to source for articles on Onchocerciasis in Cameroon.

**Results:**

Our appraisal of the literature suggests that Onchocerciasis poses a significant health and economic burden in Cameroon. A composite of factors contribute to the challenge of containing and eradicating Onchocerciasis in Cameroon and include: continuous transmission of the disease; non-compliance to mass drug administration; inability of health care providers (HCPs) to adequately diagnose the disease; limited access of most individuals in endemic zones to annual preventive chemotherapy and inadequate population education on simple and practical measures to prevent the disease. More robust population-based epidemiologic studies are needed to better quantify the current disease burden and consequently guide intervention strategies for complete disease eradication.

**Conclusion:**

Onchocerciasis is still a neglected tropical disease (NTD) in Cameroon and urgently demands a need for intensification and probably modification of some strategies in the current onchocerciasis elimination program. Control of the disease will contribute to achievement of the corresponding Sustainable Development Goals (SDGs) quota.

## Background

Onchocerciasis is a severe disabling disease with significant morbidity in humans due to its debilitating clinical features [1, 2]. The socioeconomic implications of this disease cannot be overemphasized as the disease burden has significant consequences on individuals, communities and country governments as a whole [3–5]. Onchocerciasis is considered to be a neglected tropical disease (NTD); and an estimated 99% of all cases of onchocerciasis are reported to be from Sub-Saharan Africa with over 18 million people suffering from the disease [4, 6]. According to the World Health Organization (WHO), 6.5 million people who have the disease suffer from dermatologic manifestations and 270,000 are blind [4]. The situation in Cameroon is particularly worrisome as studies have shown that over 60% of the rural population is at risk of infection with the disease [6] likely suggesting gaps in public health policies to combat this NTD [7]. Despite efforts at eradication of the disease through annual distribution of Ivermectin, some onchocerciasis-endemic regions in Cameroon still have a high prevalence of the disease; with recent studies reporting a prevalence of 40% in districts in the West region [8]. In this report, we discuss the merits and demerits of the current health policies aimed at eliminating this NTD in Cameroon.

## Methods

Our review was guided by the following strategy. We searched MEDLINE via PubMed from inception to September 2016 to identify studies on onchocerciasis in Cameroon. The following key words were used: “neglected tropical diseases”, “disease of poverty”, “onchocerciasis”, “filariaisis”, “lymphatic filariais”, “river blindness”, “elephantiasis”, “Onchocerca volvulus”, “black fly”, “simulium species”, and combined with “Cameroon”. Reference lists of all articles included were similarly searched for potential relevant articles. Our search extended to include abstracts from conference proceedings, book chapters and documents from organizations such as WHO, and the United States Centers for Disease Control and Prevention (CDC). The local Onchocerciasis control program from the Ministry of Public Health of Cameroon was also accessed for relevant data. Potentially eligible articles and documents were further screened to obtain data regarding the epidemiology and burden of Onchocerciasis (prevalence, incidence, complications/ morbidity and mortality). We also sourced for data on the economic burden of the disease to health care systems globally and Cameroon specifically. The ensuing sections of the review thus provide a synthesis of a myriad of significant literature with varied epidemiologic study designs regarding onchocerciasis in the context of, and appraising the current NTD – Onchocerciasis control program in Cameroon.

## Results and Discussion

### Burden of onchocerciasis in Cameroon

#### Disability adjusted life year (DALY) estimates due to onchocerciasis

Globally, there has been a significant decrease in the disease-burden of Onchocerciasis with the DALYs per hundred thousand decreasing from 1441.6 in 2005 to 1135.7 in 2015 [9]. However, in a country with a double disease burden like Cameroon, onchoceriasis contributes up to 0.63% of the total DALYs with an annual percentage change of −3.55% [10]. The situation is worse amongst those aged 5–14 years with the NTD consisting of 1.32% of their total DALYs [10]. The burden of the disease remains a major public health challenge for most especially endemic African countries. Coffeng and colleagues, in their updated estimates of the health impact of African Programme for Onchocerciasis Control (APOC) suggested, that the program obviated about 19 million DALYs through 2015, thereby significantly reducing the economic burden of the affection compared to previous estimates [11]. However, despite this great positive economic impact of the APOC, they proposed that the true burden of onchocercal disease was more than presented in their estimates since they failed to consider the influence of other sequela linked with onchocerciasis like epilepsy [12], disfiguring skin disease and the head-nodding syndrome which has been shown to have a significant association with the disease especially in Sub-Saharan Africa [13–15]. Though Cameroon follows the global trend in the reduction of the burden of the disease, significant effort is still warranted to achieve disease eradication.

#### Epidemiology, prevalence and trends in Cameroon

Onchocerciasis is endemic in several regions in Cameroon. Despite several years of mass distribution of Ivermectin, many authors have suggested a decrease in the prevalence of the disease but a probable non-interruption of transmission of the disease [6, 16, 17]. This seems to be a significant pitfall towards elimination and eradication of the disease. In north Cameroon, after 17 years of preventive chemotherapy, researchers in 2010 found a prevalence of positive microfilaria skin snips of 4.8 and 13.5% of palpable nodules amongst adults in the community. Furthermore, 5.5% were observed to have microfilariae in the anterior chamber of the eye [16]. Similarly, in the Western region of the country, the prevalence of microfilaria in the adult population decreased from 68.7 to 11.4% and nodule prevalence also decreased from 65.9 to 12.1% after 15 years of administration of preventive chemotherapy [17]. These values still suggest though there has been significant reduction, transmission still appears to be ongoing with significantly high infective rates [17]. In the North-west of the country, lower but significant prevalence were obtained after 7 years of preventive chemotherapy with 3.5% of the population having dermatologic manifestations of the disease and 3.7% had palpable nodules in a region where 45% of the population were infected prior to preventive chemotherapy [4].

#### Morbidity and complications of onchocerciasis

The disabling clinical features of onchocerciasis range from severe skin changes to eventual blindness [7]. Individuals affected with onchocerciasis eventually become burdens to their communities. This in part is due to the fact that they may not be able to take part in income generating activities with consequent dependence on other community members, and a vicious cycle of poverty and disease [7]. In Cameroon, the morbidity due to this disease cannot be understated, with some studies showing that over 67.5% of some populations have visual impairment which could be attributed to onchocerciasis [4].

#### Management and prevention of onchocerciasis

Onchocerciasis especially in resource-limited settings is usually diagnosed from a history of body itches, palpation of nodules on the skin and a skin snip observed under a microscope for evidence of larvae of *O. volvulus.* Other diagnostic techniques involve observing the anterior chamber of the eye for larvae or characteristic lesions using a slit-lamp examination, and anti-body tests. In Cameroon, diagnosis is mostly done by a clinical history and obtaining a skin snip [9]. Despite the high specificity of the skin snip testing, low sensitivity values ranging from 28.6 to 75.6% [18] make it an inadequate diagnostic test for a country aiming for eradication of the disease due to the number of individuals who may be missed especially in the early phase of the disease [18]. Also, this test requires a high level of technical training, a resource which is limited in the inaccessible regions of the country. In addition, the test requires a significant amount of time to perform. The opportunity cost of the technicians have to be considered as this time could be spent doing other cost-effective interventions for the community.

The standard treatment for onchocerciasis is one dose of 150 μg/kg of Ivermectin given orally every 6 to 12 months [19].

There are several basic preventive measures which could help prevent onchocerciasis. These preventive measures include: wearing long dresses especially in the evenings, keeping their immediate environments free from bushes, avoiding settlement in swampy areas and those with fast flowing rivers and streams [7]. Also, the population should be encouraged to have good health-seeking behaviors to allow for early detection of the disease and hence prevent severe concomitant complications like blindness [7].

### Onchocerciasis and the NTD control program in Cameroon

#### Overview of the NTD program

Due to the consequences of onchocerciasis on individuals and communities, several governments have strived towards the elimination of the disease especially as its elimination and control was one of the targets of the Millennium Development Goals (MDGs). The health burden of onchocerciasis is associated with the nonattainment of the MDGs: 1, 2, 5 and 6 [3]. Due to the abandonment of fertile lands from fear of the disease, several communities lived in poverty (goal 1). In addition, incapacitating skin disease and subsequent blindness of adult victims lead to them not being able to provide for their children; sending them to school then became even more laborious (goal 2). Also, onchocerciasis causes severe skin conditions which significantly affects maternal health and even prevents them from breastfeeding their newborns (goal 5). Finally, onchocerciasis is a neglected tropical disease which had to be controlled and eliminated (goal 6) [3]. In Cameroon, the inability to control the NTDs like onchocerciasis coupled with other factors like the ever increasing maternal mortality ratio and poverty levels [7, 20]; could account for the failure to achieve the above MDGs. With the advent of the Sustainable Development Goals (SDGs) [21] there is need for a revamp of health policies to ensure that Cameroon does not fail to achieve these goals too.

Most Latin American countries have used expansive programs to achieve the objective of elimination of onchocerciasis; with Colombia, Guatemala, Mexico, Ecuador and Brazil successfully achieving virtual eradication of onchocerciasis [7, 22]. The use of the Onchocerciasis Elimination Program in the Americas (OEPA) which combined vector control and annual treatment using Ivermectin was responsible for this exceptional achievement in the Americas [22]. However, using this program as a model has still seen several West African countries struggle with the elimination of the disease. The APOC modelled after the OEPA uses biannual distribution of Ivermectin to vulnerable populations but does not include vector control due to its relative non feasibility and reported cost-ineffectiveness [3, 22]. In Cameroon, the APOC works with the national Neglected Tropical Diseases Program (NTDP) which was created to eliminate the disease. The NTDP uses trained non-medical personnel mostly comprising of community volunteers to act as Community-directed Distributors (CDD) of Ivermectin throughout the national territory.

The use of this program has its merits and demerits. Firstly, the provision of free preventive chemotherapy (Ivermectin) is easy to be administered in a mass-scale, and hence, many individuals can have access to this treatment at no cost. This has great positive consequences on overall health expenditure. Furthermore, the use of CDD to administer this treatment to communities is quite effective; in populations where there is a general mistrust for the health system [23], using trained community members to provide preventive chemotherapy to members of their community could be quite helpful as people (in such areas) would feel relatively comfortable receiving drugs from fellow community members rather than from “strangers”.

However this program has several weaknesses. CDDs do not visit remote and inaccessible areas hence the people in such regions do not receive annual treatment. This highlights the fact that several regions in the country do not have access to the NTDP [7] even though, they are endemic to onchocerciasis. To ensure the success of the program, accessibility to most remote regions in the country has to be improved. This potentially calls to question whether the number of CDDs is sufficient as most of the time; only 2 CDDs are available to cover several large villages and zones. In addition, there is the problem of compliance to preventive chemotherapy even in regions which benefit from the availability of CDDs. Studies have shown low compliance to treatment amongst populations largely due to distrust of the drug and side effects [24]. In some areas, despite about 65% coverage by CDDs; over a quarter of these covered populations do not comply with the treatment [24]. This problem could potentially be averted by ensuring that CDDs consider amongst others, including and emphasizing on health education and promotion activities as their current role seems to be limited to: obtaining treatment from nearby health facilities; distribution of treatment; taking census; recording of side effects; treating minor side effects and referring major ones to more competent health facilities [25].

Furthermore, most HCPs (Health care providers) in endemic and remote zones somewhat fail to diagnose onchocerciasis either due to lack of sufficient training; absence of a highly sensitive diagnostic test or inadequate knowledge [7]. If HCPs could effectively diagnose the disease, they could easily identify and provide data necessary for mapping out zones that are at risk and endemic to the disease. This would inform the necessary public health authorities of the likely extent of spread of the disease so that the NTDP could be intensified and expanded to those areas. This drawback in diagnosing onchocerciasis coupled with the lack of active surveillance of the disease leads to non-identification of endemic zones where the NTDP needs to be addressed. Also, the lack of vector control in West Africa may be a significant pitfall in the fight against onchocerciasis. In the Americas, annual distribution of Ivermectin was coupled with vector control [22]. The success of these two programs was evident in the virtual elimination of the disease in most of the concerned countries. Vector control uses environmentally safe insecticides to kill the larvae of the Simulium fly (black fly) which is responsible for the transmission of onchocerciasis. This breaks an important chain in the life cycle of the ‘black fly’; hence elimination of the parasite is achievable. However, in endemic zones where vector control is not performed, despite annual treatment with Ivermectin, there is always the risk of reinfection as the Black fly continues reproduction hence the capacity to persistently transmit the parasite. Therefore in such regions like in Cameroon, control of the spread of the disease is achievable but achieving elimination remains doubtful.

Onchocerciasis is directly and indirectly implicated in the SDGs 1, 2, 3 and 4. As highlighted above, several individuals are bound to live in poverty due to inability to farm fertile but swampy lands (Goal 1 and 2); onchocerciasis remains endemic in some areas in Cameroon (Goal 3) and parents suffering from the disabling conditions of the disease may not be able to send their children to school (Goal 4).

In Cameroon, there has been a significant improvement in reducing the overall prevalence of onchocerciasis but a proportion of the population remain infective ensuring that transmission rates remain high. This represents a significant pitfall towards eradication of the disease and achievement of the SDGs as a whole.

Thus to aid in the achievement of the SDGs (and curb this huge burden related with Onchocercal disease), we greatly suggest a relook into the onchocerciasis control program in Cameroon by public health (and policy) authorities as proposed above to ensure a high level of effectiveness in the fight to control and eliminate the disease.

## Conclusion

Despite progress made in reducing the prevalence of onchocerciasis in Cameroon through preventive chemotherapy, continuous transmission remains a problem largely due to problems with compliance. Health policies that could help salvage this situation include: training more and improving accessibility of CDDs to remote/enclaved areas thus intensifying annual preventive chemotherapy; increasing the number of CDDs available in the NTDP; providing continuous medical education programs to HCPs to improve the recognition of the disease as well as to aid in the identification of high risk zones; encouraging mass health promotion campaigns especially in remote areas to inform populations about preventive measures and the need for compliance to preventive chemotherapy and finally reconsidering the use of vector control in highly endemic areas in an attempt to achieve elimination of the disease.

### Key messages

Onchocerciasis is a Neglected Tropical Disease which is endemic in Cameroon.

The nonattainment of the Millennium Development Goals could be blamed partly on the failure to achieve specific targets like the elimination of Onchocerciasis.

In order to attain the Sustainable Development Goals and eliminate the disease in Cameroon, we suggest some re-adaptation of the current health policies and NTD program.

## Abbreviations

APOC: African Programme for Onchocerciasis Control
CDC: Centers for disease control and prevention
CDD: Community-directed distributors
DALY: Disability adjusted life years
HCP: Health care providers
MDG: Millennium development goals
NTD: Neglected tropical disease
NTDP: Neglected tropical diseases program
OEPA: Onchocerciasis Elimination Program in the Americas
SDG: Sustainable development goals
WHO: World Health Organization

## References

[1] Taylor MJ, Hoerauf A, Bockarie M (2010). Lymphatic filariasis and onchocerciasis. Lancet.

[2] Little MP, Basanez MG, Breitling LP, Boatin BA, Alley ES (2004). Incidence of blindness during the onchocerciasis control programme in western Africa, 1971–2002. J Infect Dis.

[3] Bush S. Elimination of onchocerciasis; Ten-year strategic fast tracking plan in Sightsavers supported countries 2011 – 2021. West Sussex: Sightsavers; 2011.

[4] World Health Organisation. Onchocerciasis - river blindness Geneva: World Health Organisation; 2016. Available from: http://www.who.int/mediacentre/factsheets/fs095/en/. Accessed 3 Mar 2016.

[5] Centers for Disease Control and Prevention. Parasites - Onchocerciasis (also known as River Blindness) Atlanta: Centers for Disease Control and Prevention; 2013. Available from: https://www.cdc.gov/parasites/onchocerciasis/epi.html. Accessed 3 Mar 2016.

[6] Kamga HL, Shey DN, Assob JC, Njunda AL, Nde Fon P, Njem PK (2011). Prevalence of onchocerciasis in the Fundong Health District, Cameroon after 6 years of continuous community-directed treatment with ivermectin. Pan Afr Med J.

[7] Njim T, Ngum JN, Aminde LN (2015). Cutaneous onchocerciasis in Dumbu, a pastoral area in the North-West region of Cameroon: diagnostic challenge and socio-economic implications. Pan Afr Med J.

[8] Senyonjo L, Oye J, Bakajika D, Biholong B, Tekle A, Boakye D (2016). Factors associated with ivermectin non-compliance and its potential role in sustaining onchocerca volvulus transmission in the West Region of Cameroon. PLoS Negl Trop Dis.

[9] GBD 2015 DALYs and HALE Collaborators (2016). Global, regional, and national disability-adjusted life-years (DALYs) for 315 diseases and injuries and healthy life expectancy (HALE), 1990–2015: a systematic analysis for the global burden of disease study 2015. Lancet.

[10] Institute for Health Metrics and Evaluation. Global Burden of Disease 2015 University of Washington: Institute for Health Metrics and Evaluation; 2016 [

[11] Coffeng LE, Stolk WA, Zoure HG, Veerman JL, Agblewonu KB, Murdoch ME (2014). African programme for onchocerciasis control 1995–2015: updated health impact estimates based on new disability weights. PLoS Negl Trop Dis.

[12] Pion SD, Kaiser C, Boutros-Toni F, Cournil A, Taylor MM, Meredith SE (2009). Epilepsy in onchocerciasis endemic areas: systematic review and meta-analysis of population-based surveys. PLoS Negl Trop Dis.

[13] Williams SCP (2012). Nodding syndrome leaves baffled scientists shaking their heads. Nat Med.

[14] Winkler AS, Friedrich K, Konig R, Meindl M, Helbok R, Unterberger I (2008). The head nodding syndrome--clinical classification and possible causes. Epilepsia.

[15] Idro R, Opar B, Wamala J, Abbo C, Onzivua S, Mwaka DA (2016). Is nodding syndrome an onchocerca volvulus-induced neuroinflammatory disorder? Uganda’s story of research in understanding the disease. Int J Infect Dis.

[16] Katabarwa MN, Eyamba A, Nwane P, Enyong P, Yaya S, Baldiagai J (2011). Seventeen years of annual distribution of ivermectin has not interrupted onchocerciasis transmission in North Region, Cameroon. Am J Trop Med Hyg.

[17] Katabarwa MN, Eyamba A, Nwane P, Enyong P, Kamgno J, Kuete T (2013). Fifteen years of annual mass treatment of onchocerciasis with ivermectin have not interrupted transmission in the west region of cameroon. J Parasitol Res.

[18] Thiele EA, Cama VA, Lakwo T, Mekasha S, Abanyie F, Sleshi M (2016). Detection of onchocerca volvulus in skin snips by microscopy and real-time polymerase chain reaction: implications for monitoring and evaluation activities. Am J Trop Med Hyg.

[19] Udall DN (2007). Recent updates on onchocerciasis: diagnosis and treatment. Clin Infect Dis.

[20] Edie GE, Obinchemti TE, Tamufor EN, Njie MM, Njamen TN, Achidi EA (2015). Perceptions of antenatal care services by pregnant women attending government health centers in the Buea Health District, Cameroon: a cross sectional study. Pan Afr Med J.

[21] Williams E (2013). An equitable challenge: when sustainable development goals set the post-2015 agenda. Aust N Z J Public Health.

[22] Thylefors B (2004). Eliminating onchocerciasis as a public health problem. Trop Med Int Health.

[23] Njim T, Aminde LN, Feteh FV, Ngum JN, Moustapha CA (2015). Measles outbreak in a poorly vaccinated region in Cameroon: a case series study, public health challenges and recommendations. Pan Afr Med J.

[24] Brieger WR, Okeibunor JC, Abiose AO, Wanji S, Elhassan E, Ndyomugyenyi R (2011). Compliance with eight years of annual ivermectin treatment of onchocerciasis in Cameroon and Nigeria. Parasit Vectors.

[25] Vouking MZ, Tamo VC, Tadenfok CN (2015). Contribution and performance of female community-directed distributors in the treatment of onchocerciasis with ivermectin in Sub-Saharan Africa: a systematic review. Pan Afr Med J.

